# Lung cancer as a predominant feature in a patient with Peutz–Jeghers syndrome: Case report

**DOI:** 10.1111/1759-7714.14447

**Published:** 2022-05-11

**Authors:** Florentia Fostira, Elena Fountzilas, Kyriaki Papadopoulou, Theodoros Karaiskos, Ourania Mpatsi, Nikoleta Pastelli, Giannis Mountzios, Irene Konstantopoulou, George Fountzilas

**Affiliations:** ^1^ Molecular Diagnostics Laboratory InRASTES, National Centre for Scientific Research Demokritos Athens Greece; ^2^ Second Department of Medical Oncology Euromedica General Clinic of Thessaloniki Thessaloniki Greece; ^3^ European University Cyprus German Oncology Center Limassol Cyprus; ^4^ Laboratory of Molecular Oncology Hellenic Foundation for Cancer Research/Aristotle University of Thessaloniki Thessaloniki Greece; ^5^ Cardiothoracic Surgery Department Papanikolaou Hospital Thessaloniki Greece; ^6^ Pathology Department Papageorgiou Hospital Thessaloniki Greece; ^7^ Department of Surgical Pathology Papanikolaou Hospital Thessaloniki Greece; ^8^ Second Oncology Department and Clinical Trials Unit Henry Dunant Hospital Athens Greece; ^9^ Department of Medical Oncology German Oncology Center Limassol Cyprus

**Keywords:** case report, immunotherapy, lung cancer, Peutz–Jeghers syndrome, STK11

## Abstract

Peutz–Jeghers syndrome (PJS) is characterized by mucocutaneous pigmentation and gastrointestinal hamartomatous polyposis, which can lead to intussusception. PJS patients face high lifetime risks for various cancer types, with the majority of patients being diagnosed with tumors along the gastrointestinal tract. Herein, we present the case of a 34‐year‐old man who carried a germline *STK11* pathogenic variant, while lacking the cardinal features of PJS syndrome. Interestingly, he was diagnosed with lung adenocarcinoma despite being a never‐smoker. Tumor testing revealed clinically relevant molecular alterations, including the known germline pathogenic variant *STK11*, a *KRAS* somatic pathogenic variant, and *FGFR3* gene amplification. Treatment with standard chemotherapy and immunotherapy did not have a clinical benefit. Due to clinical deterioration, the patient deceased 18 months after his initial diagnosis prior to having the chance for targeted therapy. Identification of rare hereditary cancer syndromes and the respective presence of tumor biomarkers can provide important alternatives to targeted treatments, including immunotherapy in patients with tumors unresponsive to conventional treatment protocols. This case highlights that although only a small proportion of lung cancer diagnoses will be due to hereditary predisposition, *STK11* germline carriers should be under close surveillance for early detection of lung cancer.

## Graphical abstract

Peutz–Jeghers syndrome is a rare syndrome associated with loss‐of‐function *STK11* variants and is one of the few reasons for hereditary predisposition to lung cancer. Prompt identification of *STK11* carriers can provide options for targeted therapies.

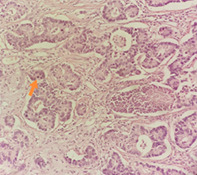

## INTRODUCTION

Peutz–Jeghers syndrome (PJS) is a rare hereditary syndrome characterized by mucocutaneous pigmentation along with gastrointestinal hamartomatous polyposis and increased risk for multiple types of cancer. Malignancies of the gastrointestinal tract, i.e. gastric, small bowel, colon, rectum, and pancreas, followed by breast, ovary, and testicular cancers are the most frequently diagnosed malignancies among patients with PJS. Due to their elevated lifetime cancer risks, estimated to be around 85%,^1^, ^2^ individuals with PJS should be offered multidisciplinary care and follow specialized screening protocols. Germline pathogenic *STK11/LKB1* variants account for most individuals with a PJS clinical diagnosis.

Lung cancer, although described in the PJS tumor spectrum, is a relatively rare entity, with men and women with PJS facing a 13% and 1% risk, respectively, for diagnosis by the age of 60 years.^2^ Overall, lung cancer is a tumor type mostly associated with environmental factors, prominently tobacco smoking, with hereditary predisposition identified in a very small subset of cases.

Herein, we present an unusual case of a young, never‐smoker, PJS patient, who lacked the characteristic PJS features, with lung adenocarcinoma being his prominent diagnosis.

## CASE PRESENTATION

**FIGURE 1:**
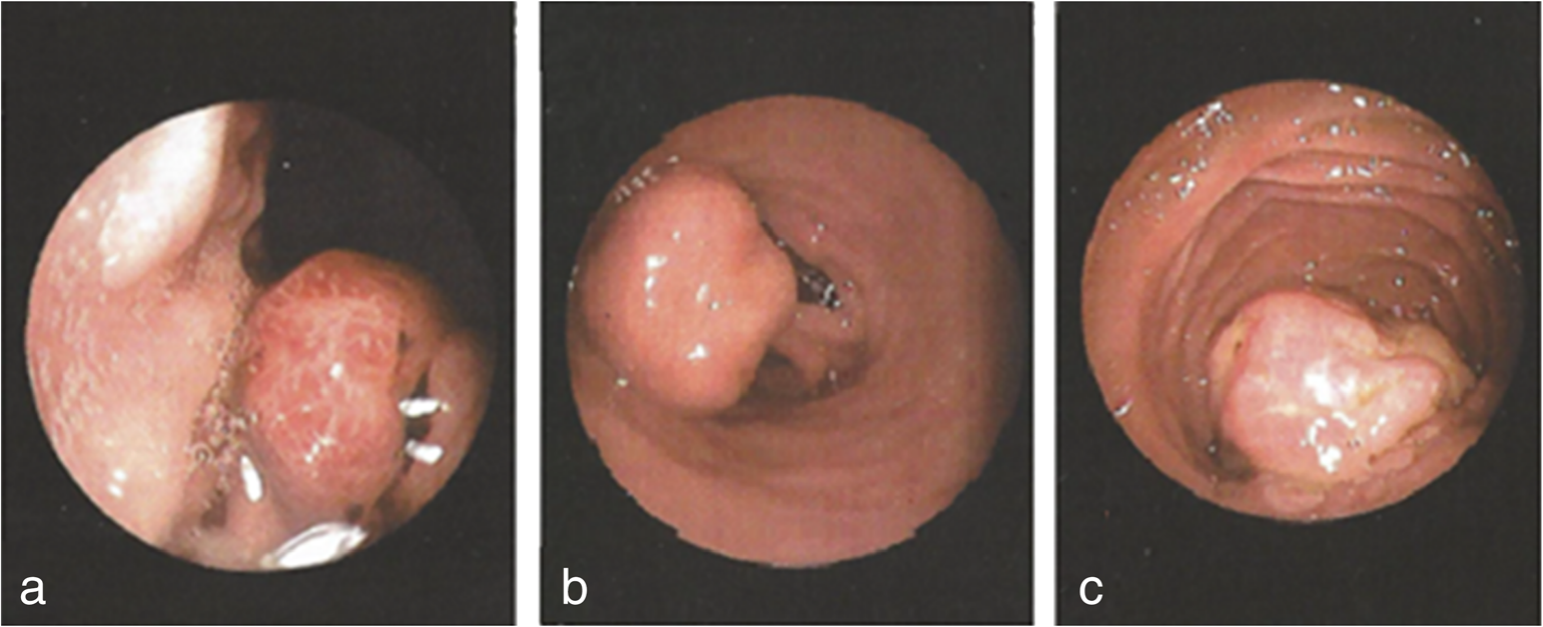
Peutz–Jeghers syndrome polyps identified during upper gastrointestinal endoscopy: (a) gastric body sessile lesion, (b) duodenal bulb pedunculated lesion, and (c) sessile lesion involving the ampulla

In February 2017, a 34‐year‐old man with a medical history of schizophrenia presented with anemia and underwent diagnostic gastroscopy. One and two nondysplactic hyperplastic polyps were identified in his stomach and duodenum, respectively (Figure 1). In June 2017, colonoscopy revealed a tubular adenoma with low‐grade dysplasia in the sigmoid, while a follow‐up colonoscopy revealed two additional polyps.

**FIGURE 2:**
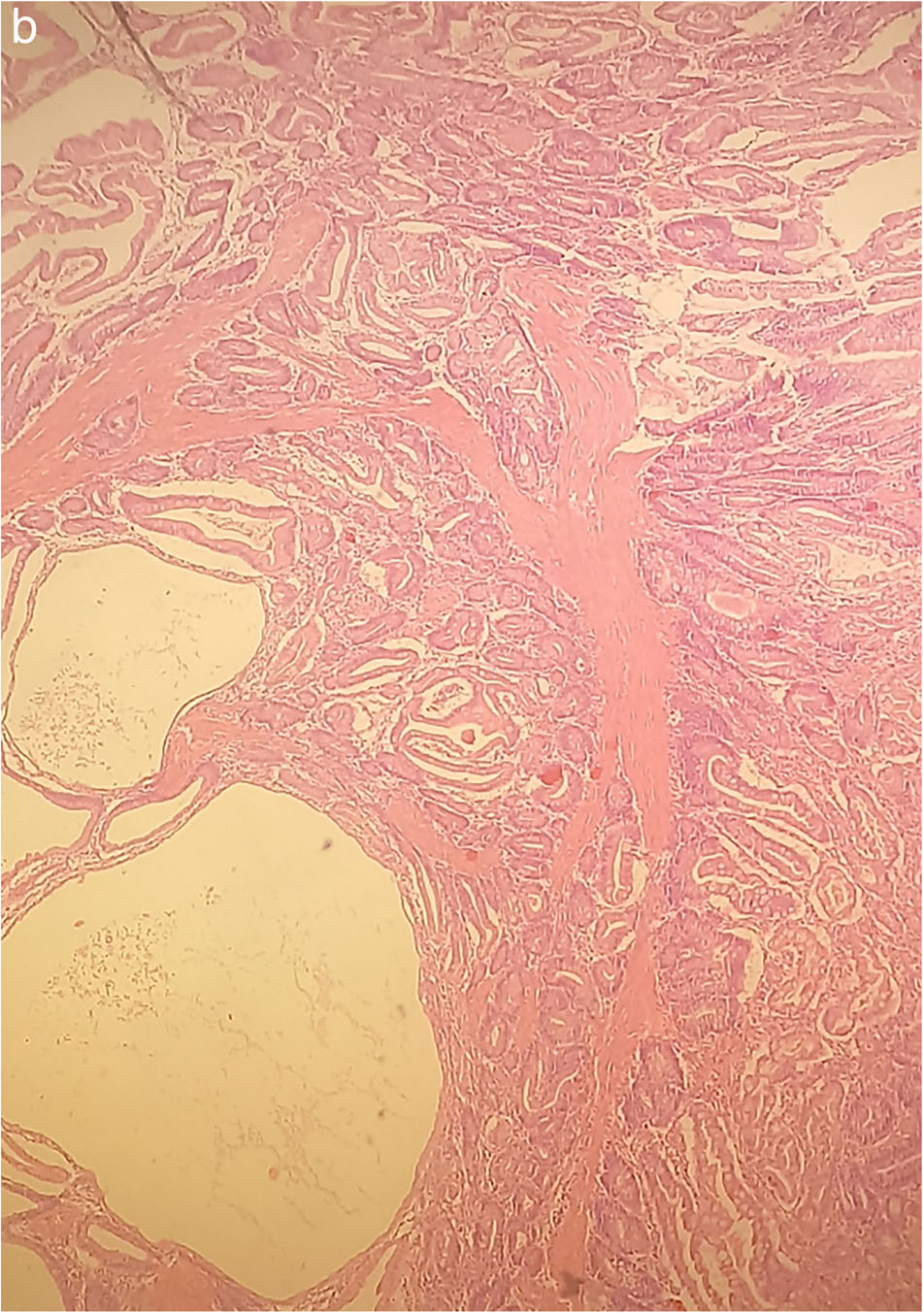
Hematoxylin and eosin staining of a hamartomatous polyp, where the central core of branching smooth muscle, typical of Peutz–Jeghers syndrome, is presented

**FIGURE 3:**
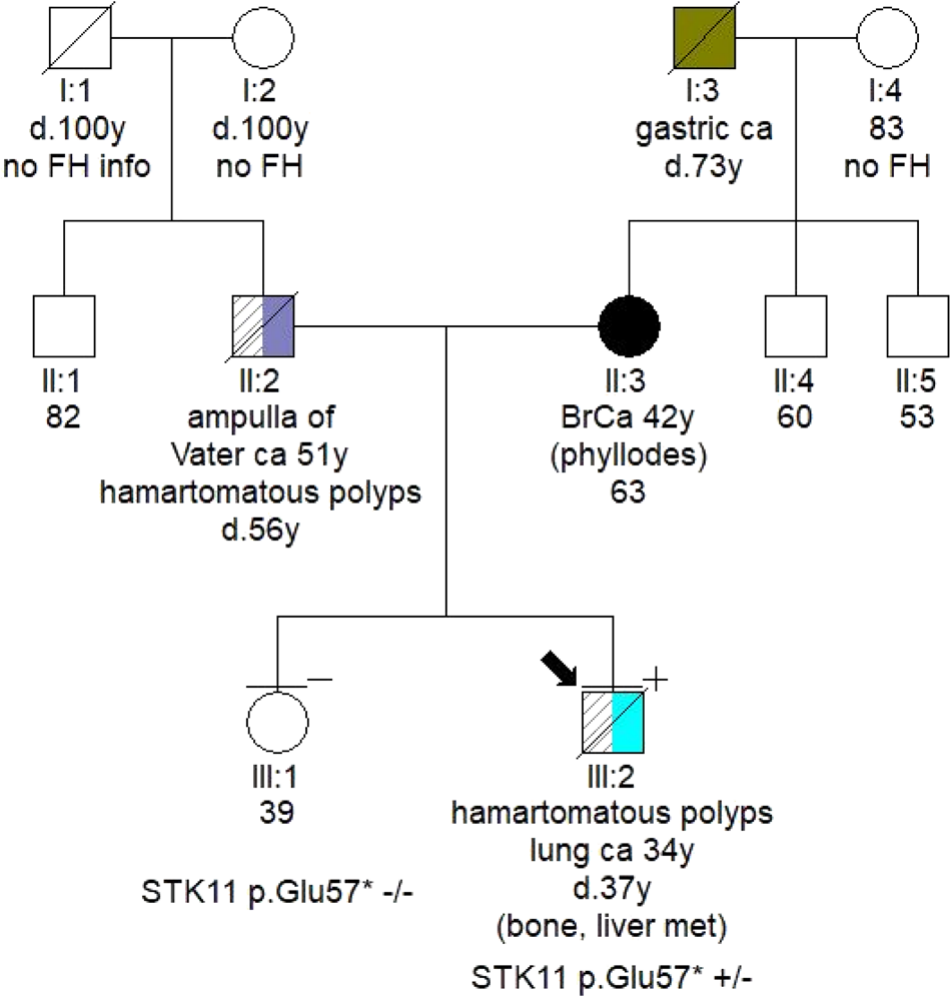
Detailed family pedigree. The patient's father had been diagnosed with carcinoma of the ampulla of Vater at the age of 51 years. Subsequent endoscopies demonstrated hamartomatous polyps in both his small intestine and jejunum. He was further diagnosed with metastatic pancreatic and small intestinal cancer, and died at the age of 56 years

In December 2017, the patient was hospitalized with small bowel obstruction due to intussusception and underwent surgical resection, followed by end‐to‐end anastomosis. Histology report described two hamartomatous polyps with a central core of branching smooth muscle, typical of PJS (Figure 2). At this point, genetic testing for *STK11* was ordered, based on recent diagnosis, and his family history. His father was diagnosed with ampulla of Vater cancer at the age of 51 years, while at subsequent endoscopies hamartomatous polyps were identified his small intestine and jejunum. He died from metastatic pancreatic cancer at the age of 56 years. A detailed pedigree is illustrated in Figure 3.

Germline testing led to the identification of a *STK11* pathogenic variant, c.169G>T, p.(Glu57Ter). The variant has an entry on ClinVar database and has been previously reported in the literature,^3^ but has not been reported in patients of Greek descent.^4^ Archival blood sample was used to verify the presence of the variant in the patient's father.

**FIGURE 4:**
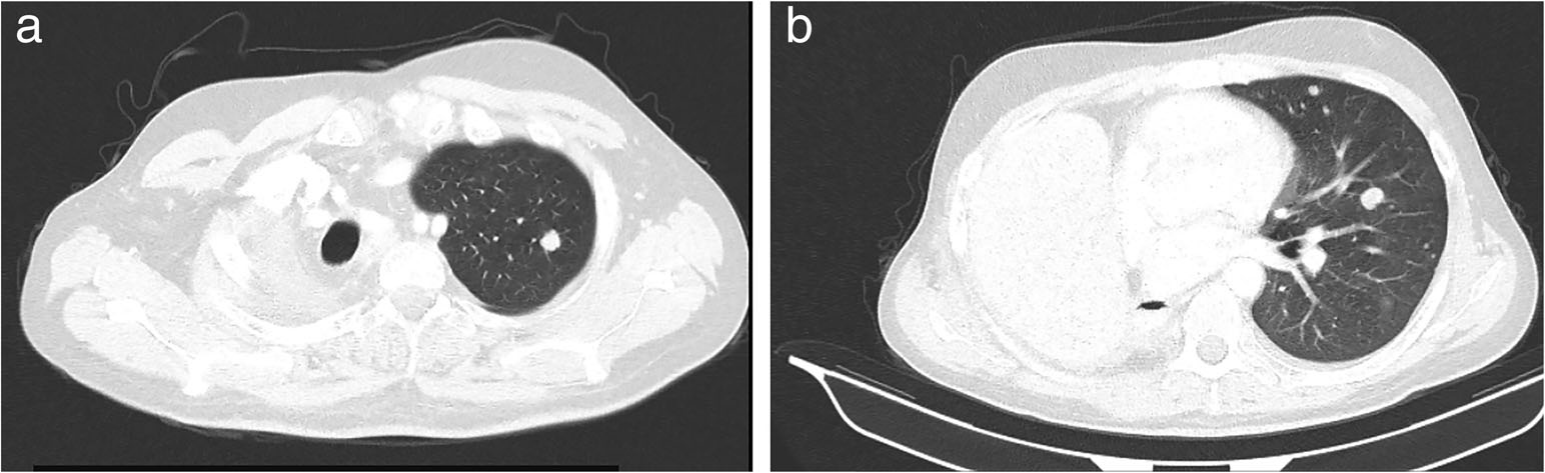
(a) Positron emission computed tomography scan at lung cancer diagnosis showing an enhanced lobulated lesion of the middle lobe of the right lung. (b) Computed tomography scan demonstrating disease progression with new pulmonary nodules in the left lung

**FIGURE 5:**
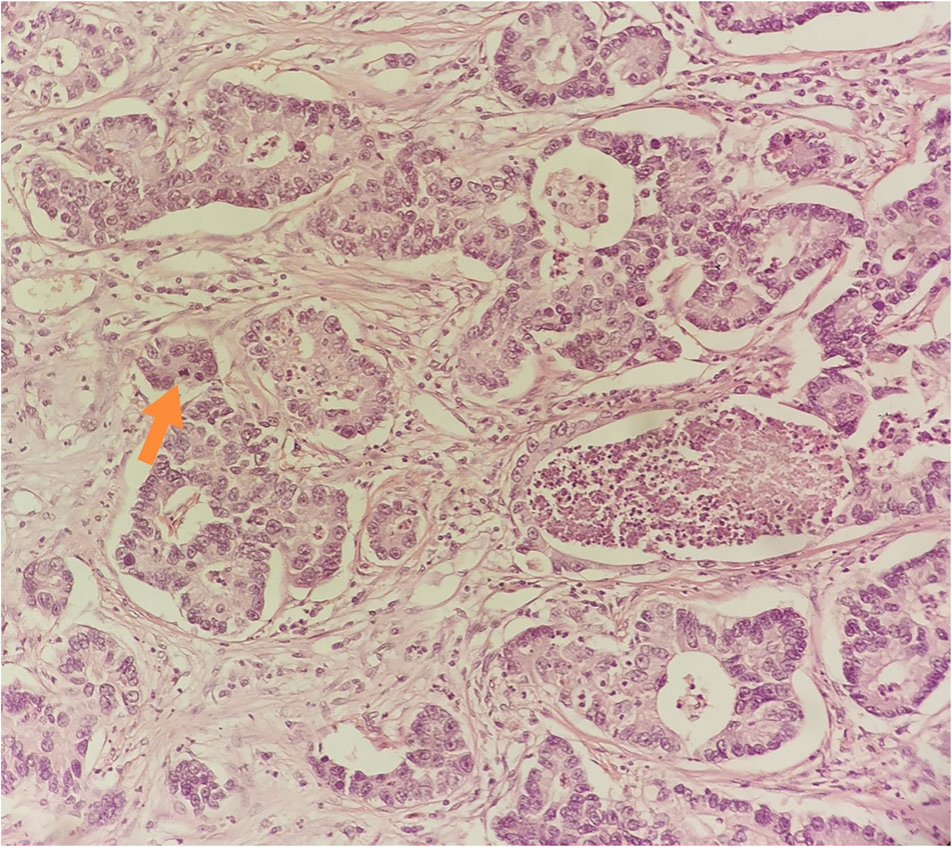
Hematoxylin and eosin staining (20×) showing cancerous glands with intraluminal necrosis and some mitotic figures

On January 2018, the patient underwent a positron emission computed tomography (CT) scan which demonstrated an enhanced lobulated lesion of the middle lobe of the right lung (Figure 4a). A subsequent CT‐guided biopsy was diagnostic for lung adenocarcinoma. The patient, who reported no history of smoking or alcohol consumption, underwent right pneumonectomy and mediastinal lymphadenectomy in June 2018. Histologic examination revealed a T2bN2 non‐small‐cell lung (NSCLC) adenocarcinoma (Figure 5). Following diagnosis, adjuvant chemotherapy with four cycles of carboplatin and paclitaxel was administered.

In December 2019, disease progression was confirmed via CT scan, with the presence of new pulmonary nodules and bone lytic lesions (Figure 4b). At this point, tumor molecular profiling was ordered to identify potential therapeutic targets, which revealed the pathogenic *STK11* variant with Variant Allele Frequence (VAF) of 74%, suggesting loss of heterozygosity at the specified position, the *KRAS* c.35G>C pathogenic variant (VAF‐19%), and *FGFR3* gene amplification (copy number gain‐5.29 copies). No other targetable molecular alterations (*EGFR* mutation or *ALK* fusions) were identified, while programmed death‐ligand 1 (PD‐L1) expression was negative.

In January 2020, first‐line treatment with carboplatin, pemetrexed, and pembrolizumab was initiated. After four cycles of treatment, CT scan documented disease progression with the development of new lung and liver metastatic lesions. Gemcitabine was administered as second‐line treatment, resulting in significant clinical improvement, with pain reduction and improved mobility. The patient completed three cycles of chemotherapy and was scheduled for restaging examinations; however, in June 2020, he presented with acute respiratory distress and despite resuscitation efforts he died due to cardiorespiratory arrest. No autopsy was performed (Figure 5).

## DISCUSSION

We herein present an unusual case of a 34‐year‐old man with PJS who lacked the characteristic pigmentation, had only a small number of the typical hamartomatous polyps, while his prominent diagnosis was an advanced stage lung adenocarcinoma. A year after his initial diagnosis, disease recurrence was observed. The patient received standard treatment based on current guidelines, but despite all therapeutic efforts he died 18 months after his initial diagnosis.

Tumor molecular profiling revealed clinically relevant molecular alterations, including the known germline pathogenic variant *STK11*, a *KRAS* pathogenic variant, and *FGFR3* gene amplification. Even though no targeted treatment was at that time available, these alterations warrant further investigation as prognostic or predictive biomarkers of response or resistance to immunotherapy and/or targeted treatments.

While somatic *STK11* pathogenic variants are frequent in lung adenocarcinomas, germline pathogenic variants are rarely identified in these patients. Inactivating *STK11* molecular alterations have been associated with decreased PD‐L1 expression, cytokine release, T‐cell tumor infiltration, and antigenicity, resulting in a “cold” tumor immune microenvironment.^5^, ^6^, ^7^ Lung cancer patients with co‐occurring mutations in *KRAS* and *STK11* have a trend towards shorter overall survival, while *STK11* mutational status does not seem to be a standalone prognostic factor.^8^

Notably, patients with *STK11/KRAS* co‐mutated tumors have significantly shorter progression‐free and overall survival, along with decreased overall response rates, after treatment with anti‐PD‐1 agents when compared to patients having *KRAS* mutations only.^9^ Interestingly, the presence of *STK11* pathogenic variants has been associated with worse clinical outcomes, in terms of survival and response rates, in patients with NSCLC who received treatment with immunotherapy combined with doublet chemotherapy.^10^

Hereditary lung cancer is rare, with germline *STK11* pathogenic variants being a significant genetic predisposing factor. We experienced the case of a patient who did not have the cardinal PJS features and was diagnosed with NSCLC bearing both germline and tumor *STK11*, along with tumor *KRAS* pathogenic variants, and who did not respond to combinational treatment with chemotherapy and immunotherapy. The identification of rare hereditary cancer syndromes, along with tumor biomarkers, can lead the way to select individualized treatments in the future.

## CONFLICT OF INTEREST

E.F.: Advisory role: LEO Pharma. Speaker fees: Roche, Pfizer, AstraZeneca. Stock ownership: GENPREX INC, Deciphera Pharmaceuticals, Inc. Travel grant: Merck, Pfizer, and K.A.M Oncology/Hematology and DEMO. G.F.: Advisory Board: Pfizer, Novartis and Roche. Honoraria: Astra Zeneca. Stock ownership: ARIAD, GENPREX, Daiichi Sankyo, RFL Holdings, FORMYCON. G.M.: Advisory and consultation fees: AstraZeneca, BMS, MSD, Roche, Takeda, Novartis, AMGEN. Travel and accommodation fees: AstraZeneca, BMS, MSD, Roche, Takeda, Novartis. PI in sponsored clinical trials: Novartis, Roche, MSD, AstraZeneca, Merck, ΒΜS, AMGEN. The other authors have no conflict of interest to declare.
